# Combination of pegylated liposomal doxorubicin (PLD) and paclitaxel in patients with advanced soft tissue sarcoma: a phase II study of the Hellenic Cooperative Oncology Group

**DOI:** 10.1038/sj.bjc.6602148

**Published:** 2004-10-19

**Authors:** D Bafaloukos, C Papadimitriou, H Linardou, G Aravantinos, P Papakostas, D Skarlos, P Kosmidis, G Fountzilas, H Gogas, C Kalofonos, A M Dimopoulos

**Affiliations:** 1 Department of Medical Oncology, Metropolitan Hospital, Ethnarhou Makariou Street, No. 9, N. Faliro, Athens 18547, Greece; 2 Department of Clinical Therapeutics, Alexandra Hospital, Athens University School of Medicine, Athens, Greece; 3 Third Department of Medical Oncology, Agii Anargiri Cancer Hospital, Athens, Greece; 4 Department of Oncology, Ippokration Hospital, Athens, Greece; 5 Second Department of Medical Oncology, Errikos Dynan Hospital, Athens, Greece; 6 Second Department of Medical Oncology, Hygeia Hospital, Athens, Greece; 7 Department of Medical Oncology, AHEPA Hospital, Aristotle University of Thessaloniki, Thessaloniki, Greece; 8 First Department of Medicine, Laikon University Hospital, Athens, Greece; 9 Oncology Section, Department of Medicine, University of Patras School of Medicine, Rio Patras

**Keywords:** advanced soft tissue sarcoma, chemotherapy, paclitaxel, liposomal doxorubicin

## Abstract

Patients with soft tissue sarcoma (STS), even after complete local disease control, often relapse locally or with distant metastases. This multicenter phase II study was conducted to evaluate the safety and efficacy of the combination of pegylated liposomal doxorubicin (PLD) and paclitaxel, as first-line treatment in patients with advanced STS. In all, 42 patients with locally advanced or metastatic STS, median age 54 years and median Eastern Cooperative Oncology Group performance status (PS) 1 were treated with PLD 45 mg m^−2^ and paclitaxel 150 mg m^−2^, every 28 days for a total of six cycles. Histological types included mainly leiomyosarcomas (43%), malignant fibrous histiocytomas (14%) and liposarcomas (12%). At study entry, 69% of patients had distant metastases. Overall response rate was 16%, including one complete (CR 2%) and six partial responses (PRs 14%), while an additional 14 patients had disease stabilization (SD 33%). At median follow-up 41.5 months, median time to progression (TTP) was 5.7 months with median overall survival (OS) 13.2 months. Grade 3–4 toxicities included neutropenia (17%), anaemia (15%), neurotoxicity (5%) and palmar–plantar erythrodysesthesia (9%). There were no treatment-related deaths. The combination of PLD and paclitaxel is a safe and well-tolerated regimen demonstrating modest efficacy as first-line treatment in patients with advanced STS.

Advanced soft tissue sarcomas (STSs) remain a challenging malignancy to treat, not only because of their high clinicopathological heterogeneity but also because of their limited responsiveness to most chemotherapeutic agents. Almost 50% of patients who achieve good local control will eventually relapse distantly. Patients with advanced or metastatic STS have a dismal prognosis, with median survival less than 1 year (Sarcoma Meta-analysis Collaboration, 1997).

A large number of agents have been evaluated for the treatment of advanced STS over the last 30 years, but only a handful have shown definitive activity (Spira and Ettinger, 2002). These include doxorubicin, ifosfamide and dacarbazine. Doxorubicin and ifosfamide are considered the cornerstones of treatment for advanced STS, consistently producing responses as monotherapy in more than 20% of previously untreated patients (Pinedo and Kenis, 1977). Most studies have demonstrated a clear dose–response relationship for both of these drugs (O'Bryan *et al*, 1977, Antman *et al*, 1990). Combinations of the active drugs have shown improved response rates, but are often limited by increased toxicity (Benjamin, 1987). Importantly, no benefit in overall survival (OS) has yet been achieved in comparison with single-agent doxorubicin (Edmonson *et al*, 1993, Santoro *et al*, 1995). The demonstration of a dose–response curve for doxorubicin, led to several investigational attempts to maximise the therapeutic index of this drug and its combinations. The development of growth factor support made it feasible to elevate the doxorubicin dose to 75 mg m^−2^ in combinations with ifosfamide, achieving much higher response rates (Steward *et al*, 1993). However, numerous reports of anthracycline–ifosfamide combinations in STS showed that higher doses yielded higher responses, but also more toxicity, and little, if any, change in OS (De Pas *et al*, 1998; Patel *et al*, 1998).

These results clearly indicate the need for novel approaches to the treatment of advanced STS (Santoro, 1999). In an attempt to increase the antitumour activity of doxorubicin and reduce its dose-limiting toxicities, new formulations have been developed. Pegylated liposomal doxorubicin (PLD, CAELYX™) is a liposomal formulation of doxorubicin, sterically stabilised by coupling segments of polyethylene glycol (PEG) onto the liposomal surface. Thus higher doses can be delivered safely and more efficiently (Lasic, 1996; Stewart and Harrington, 1997). This encapsulation of doxorubicin has been shown to reduce the nonspecific drug delivery to normal tissues, and the high peak plasma levels of free drug, both of which contribute to toxicity. The delivery of doxorubicin to tumour sites with improved specificity may be possible. Pegylated liposomal doxorubicin was shown to have equivalent activity to doxorubicin and an improved toxicity profile in experimental models and clinical studies (Gabizon *et al*, 1994; Uziely *et al*, 1995).

Soft tissue sarcoma seems the ideal target for PLD, since the parental drug doxorubicin is the mainstay of treatment and a clear dose–response relationship is recognised. Several published studies addressing the activity of PLD in STS are now available; unfortunately not all have comparable results (Casper *et al*, 1997; Chidiac *et al*, 2000). There are some 10 phase I/II trials reported with single agent use of PLD in STS including a recent randomised phase II study comparing PLD with standard doxorubicin (Judson *et al*, 2001). In general, these studies indicate that PLD is well tolerated with activity demonstrated in most cases. Recently the data from eight initial studies were reviewed, concluding that the activity of PLD in STS is a potential reality (Toma *et al*, 2000). Although PLD was used with the aim to increase dose intensity of doxorubicin, the doses evaluated so far, are lower than those estimated necessary for optimal response compared to doxorubicin. However, PLD has an extremely long half-life and only comparative pharmacokinetic data can allow real comparisons of the relative dose intensities of free and encapsulated doxorubicin. Comparative data on the efficacy of the two formulations in STS can be extracted by their direct clinical comparison in the randomised EORTC phase II study (Chidiac *et al*, 2000). In this study, PLD (50 mg m^−2^ every 4 weeks) was found to have equivalent efficacy to doxorubicin (75 mg m^−2^ every 3 weeks) in patients with advanced STS, with an improved toxicity profile.

The taxanes have different mechanisms of action and they also appear to have single-agent activity in advanced STS (Casper *et al*, 1998). Docetaxel has demonstrated activity rates of 17% in pretreated patients (van Hoesel *et al*, 1994; Kostler *et al*, 2001). Initial studies with paclitaxel monotherapy showed activity, with major responses seen in eight out of nine patients with angiosarcoma (Fata *et al*, 1999). Another study using single agent paclitaxel resulted in 12.5% overall response rates (ORRs) (Balcerzak *et al*, 1995). Two studies evaluating paclitaxel in combination with either Epirubicin or doxorubicin in advanced STS, concluded that it did not increase the known activity of anthracylines, as 20% response rates were achieved (Sandler *et al*, 1998; Pivot *et al*, 2002).

The proven synergistic activity of paclitaxel with anthracyclines (Gianni *et al*, 1995), and the single-agent activity of this agent as well as the demonstrated equivalent activity of PLD to doxorubicin in advanced STS, constituted the rationale for the present study performed by the Hellenic Cooperative Oncology Group. This was a phase II study carried out to assess the efficacy and toxicity of the combination of PLD and paclitaxel as first-line treatment in patients with locally advanced or metastatic STS.

## PATIENTS AND METHODS

### Patient selection

Patients with histologically confirmed advanced STS, <75 years of age, Eastern Cooperative Oncology Group (ECOG) performance status (PS) ⩽2 and evidence of measurable or evaluable disease were eligible. No previous chemotherapy for advanced disease was allowed. Previous adjuvant or neo-adjuvant treatment with an anthracycline-containing regimen was allowed provided that there was at least a 12-month treatment-free interval. Adequate haematological, renal and hepatic functions were required (WBC >3.5 × 10^9^ l^−1^, platelets >100 × 10^9^ l^−1^, bilirubin <1.2 mg dl^−1^, SGPT, *γ*GT, alkaline phosphatase normal, creatinine <1.4 mg dl^−1^ or creatinine clearance >70 ml min^−1^). Pregnant patients or those with other malignant tumour or tumour history, except for nonmelanoma skin cancer or radically excised carcinoma *in situ* of the uterine cervix, were excluded. Furthermore, patients with an active infection or other serious medical or mental condition, which would impair their ability to receive protocol treatment, including prior allergic reactions to drugs containing cremophor, were not eligible. Exclusion criteria also included neurological disorders or pre-existing motor or sensory neurotoxicity grade ⩾2. Furthermore, gastrointestinal stromal tumours (GIST) were excluded, while all other STS histologies were allowed. The study was conducted according to the Declaration of Helsinki and the Guidelines for Good Clinical Practice. The Scientific Committee of the Hellenic Cooperative Oncology Group approved the study, and informed consent was obtained from all patients prior to study entry. Ethical approval was provided by local Institutional Review Boards in participating institutions and centrally granted by the HECOG Protocol Review Committee, according to the Group's bylaws.

### Treatment schedule

Treatment was administered on an outpatient basis. Pegylated liposomal doxorubicin of 45 mg m^−2^ was administered by an intravenous infusion over 30 min on day 1, followed by paclitaxel 150 mg m^−2^ as an intravenous infusion over 3 h, on day 1. Cycles were repeated every 28 days. All patients received standard premedication prior to paclitaxel administration, in order to prevent hypersensitivity reactions. Standard antiemetic premedication and treatment was also administered. Haematopoietic growth factors were not used prophylactically. Patients received a total of six cycles unless disease progression or unacceptable toxicity occurred.

### Dose modifications

Toxicity was evaluated before each treatment cycle according to the National Cancer Institute Common Toxicity Criteria (NCI CTC version 2.0). Chemotherapy courses were given on schedule providing that ANC was ⩾1.5 × 10^9^ l^−1^ and the platelet count ⩾100 × 10^9^ l^−1^. If ANC <1500 or PLT <100 on day 1, treatment was delayed for 1 week. If blood count did not recover after 3 weeks, the patient was considered off protocol. Both drug doses were reduced by 25% in case of grade 3–4 myelotoxicity or neutropenic fever. In case of grade 3–4 neutropenia during a chemotherapy cycle, haematopoietic growth factor (G-CSF) 5 *μ*g kg^−1^ was used prophylactically on days 7–12, for all subsequent cycles. For any major organ toxicity grade >2, treatment was discontinued. In case of symptomatic arrhythmia, AV block (except first degree), or other heart blocks, paclitaxel infusion was stopped; the patient received treatment and was taken off protocol. Furthermore, for any hypersensitivity reaction paclitaxel infusion was interrupted. Following treatment for the reaction, paclitaxel infusion was reinitiated slowly with close attendance. If a severe reaction reoccurred treatment was stopped. In case of palmar–plantar erythrodysesthesia (PPE) treatment was interrupted until resolution of symptoms. If symptoms persisted for more than 4 weeks treatment was discontinued.

### Efficacy evaluation

Baseline evaluation included a complete past medical history focusing on heart, lung, and kidney function; physical examination; computed tomography (CT) scans of the chest, abdomen and pelvis; bone scan; complete blood counts; and renal and liver function tests. Evaluation during treatment included CBC, renal and liver function tests obtained before each course. CBC was also obtained on day 14 of the first course in order to assess nadir WBC and PLT. Tumour evaluation was repeated after three courses and at completion of treatment. Dose intensity was defined as the total amount of the drug given (mg m^−2^) per week. Standard WHO criteria were used to assess response. Follow-up disease evaluation was performed at 3 monthly intervals following the end of treatment. As response was the primary end point, all responses from participating institutions were centrally reviewed by a radiologist not participating in the study. All patients were analysed for efficacy on an intention-to-treat basis. All patients receiving at least one cycle of chemotherapy were analysed for toxicity. All adverse events resulting in discontinuation of study drug were followed closely until resolution or stabilisation. Patients were followed every 3 months after the completion of treatment.

### Statistical analysis

The study was a nonrandomised, phase II study. The primary end point was objective response rate and secondary end points were OS, time to progression (TTP) and toxicity. The sample size was calculated on the assumption that a 40% response rate would be detected and the minimum acceptable response rate would be 20%. According to Simon's two-stage design, a sample of 18 patients was required in the first step. If a minimum of five responses were observed a total of 33 patients would be accrued. Thereby, if at least 11 responses occurred the probability of accepting a treatment with a real response rate of less than 20% would be 5%. On the other hand, the risk of rejecting a treatment (at the second stage) with a response rate of more than 40% would be 20%. Time to progression was calculated from the initiation of treatment to the date of progression of the disease was firstly documented (patients who discontinued their treatment for any reason or died from disease-related causes were considered, at that time, as having disease progression). Survival was calculated from initiation of treatment to the date of last contact or to the date of death. The Kaplan–Meier method was used to calculate TTP and survival curves (Kaplan and Meier, 1958). Data analysis was performed using the computerised statistical package SPSS version 10.0.

## RESULTS

### Patient characteristics

Between January 1998 and October 2001, 42 patients with locally advanced or metastatic STS were enrolled into the study. The main patient baseline characteristics are summarised in Table 1

**Table 1:** Patient characteristics

	**Patients**	
**Characteristic**	**No.**	**%**
Number of patients included in final analysis	42	
*Age (years)*		
Median (range)	54 (18–78)	

*Sex*		
Male	16	38
Female	26	62

*ECOG performance status*		
0	15	36
1	24	57
2	3	7
Median	1	

*Treatment of primary disease*		
Complete surgical excision	21	50
Postsurgical radiotherapy	15	36
Adjuvant chemotherapy	9	21

*Site of primary disease*		
Visceral	18	43
Gastrointestinal	5	12
Lung	3	7
Uterus	10	24
Extremities	8	19
Retroperitoneal	5	12
Other	11	26

*Site of metastatic disease*^a^		
Locally advanced disease	26	62
Metastatic disease	29	69
Lung	18	43
Liver	6	14
Lymph nodes	4	10
Bone	1	2
>One metastatic sites	17	40

a Percentages exceed 100% since some patients have more than one metastatic site.

ECOG=Eastern Cooperative Oncology Group.

. Patient age ranged from 18 to 78 years, with a median age of 54 years. Median ECOG PS was 1 (range, 0–2). Primary disease sites were visceral in 43% of cases (gastrointestinal tract, 12%; lungs; 7%; uterus, 24%), in extremities (19%), retroperitoneal (12%), and other (26%). Half of the patients (50%) had undergone complete surgical excision of primary disease, while 36% had received postsurgical radiotherapy and 21% had received adjuvant chemotherapy. The adjuvant regimens used were mainly anthracycline based (doxorubicin monotherapy in one case, epirubicin monotherapy in two cases, ifosfamide monotherapy in three cases and combinations of ifosfamide–doxorubicin in three cases). In all cases adjuvant chemotherapy was completed more than 1 year prior to study entry. Concerning disease extent at study entry, 31% of patients had only locally advanced disease, while 69% had distant disease (lungs, 43%; liver, 14%; lymph nodes, 10%; bones, 2%), with multiple site involvement (more than one metastatic site) in 40%. Characteristics of primary and metastatic disease are shown in Table 1. The majority of patients (67%) presented with symptomatic disease at study entry, while significant weight loss (>10%) was reported in only 5% of the patients at study entry. The most common histological types included leiomyosarcomas (43%), malignant fibrous histiocytomas (14%) and liposarcomas (12%). The primary lesion was reported as >5 cm in size in 71% of the cases, while the histological grade of the primary lesion was >2 in 30% of the cases. Details of histology, differentiation and size of primary disease are shown in Table 2

**Table 2:** Histological characteristics of primary disease

**Histological types**	***N***	**%**
Leiomyosarcoma	18	43
Malignant fibrous histiocytoma	6	14
Liposarcoma	5	12
Mixed műllerian tumour	2	5
Malignant schwannoma	2	5
Neurofibrosarcoma	2	5
Synovial sarcoma	2	5
Chondrosarcoma	1	2
Malignant mesenchymoma	1	2
Epithelioid sarcoma	1	2
Unclassified	2	5

*Grade*		
I	6	14
II	10	24
III	17	40
Unknown	9	22

*Size of primary lesion*		
⩽5 cm	7	17
>5 cm	30	71
Unknown	5	12

.

### Response and survival

From a total of 42 patients included in the analysis, six (14%) were not evaluable for response but were included as nonresponders according to an intention-to-treat analysis. One patient (2%) refused to start treatment, while five patients (12%) discontinued treatment prior to response evaluation (following the first treatment cycle, two patients discontinued due to disease progression, two more patients refused to continue due to toxicity and withdrew consent while a further patient moved to different hospital care and also withdrew consent). Seven patients achieved an objective response, for an ORR of 16% (95% CI 7–31.4), including one complete response (CR 2%, 95% CI 0.06–12.6) and six partial responses (PRs 14%, 95% CI 5.4–28.5). In addition, 14 patients had disease stabilisation (SD 33%, 95% CI 19.6–49.6), and 15 patients had progressive disease (PD 36%, 95% CI 21.6–52).

**Figure 1:**
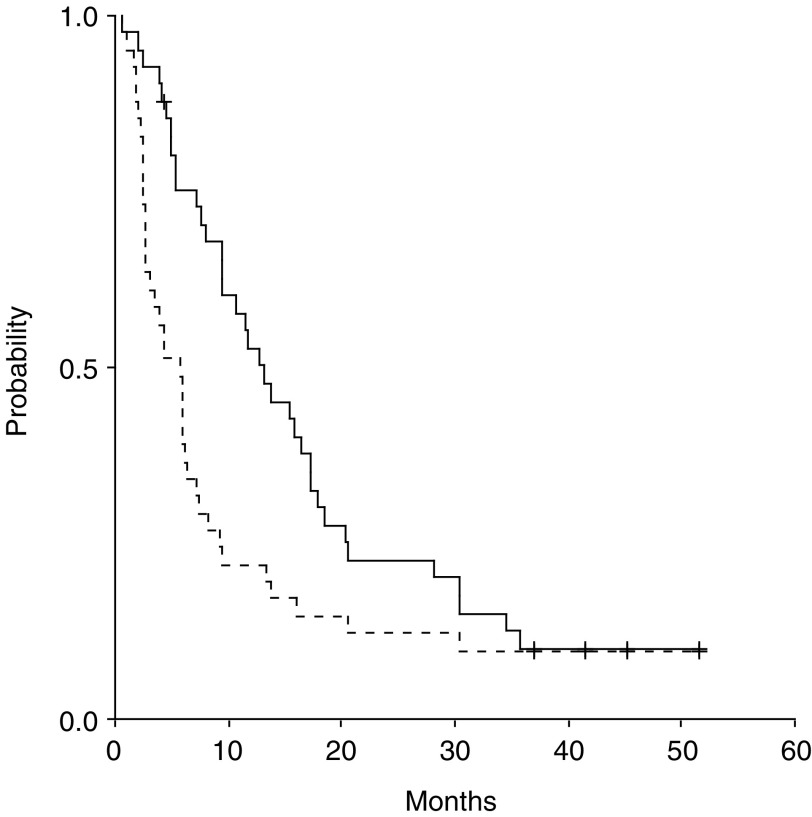
Time to progression (- - - -) and OS (—) of 42 patients with advanced STS treated with PLD/paclitaxel combination. Median Follow-up: 41.5 months, median survival: 13.2 months, events/total: 36/42, median TTP: 5.7 months.

With a median follow-up of 41.5 months (range, 0.56–51.6 months), the median TTP was 5.7 months (range, 0.56–51.6 months; 95% CI 3.06–8.4). Disease progression was reported in 36 patients. The median OS for all patients was 13.2 months (range, 0.6–51.6 months; 95% CI 8.56–17.87). Time to progression and OS curves are shown in Figure 1

.

### Study treatment and toxicity

A total of 164 treatment cycles were administered. Patients received a median number of four cycles of chemotherapy, with a range of cycles. Of the 42 patients entered, 16 patients (38%) completed treatment, while 26 patients (62%) discontinued treatment due to disease progression, toxicity, or other reasons. The majority of treatment cycles were administered with full dose of paclitaxel (90%) and full dose of PLD (82%). The median duration between cycles was 28 days (range, 21–49). Haematopoietic growth factor (G-CSF) support was required for 54% of the patients. The median relative dose intensity for paclitaxel was 0.99 (range, 0.7–1.0), and for PLD was 0.95 (range, 0.7–1.0).

All patients were evaluable for toxicity. Grade 3 haematological toxicities included neutropenia (12%), anaemia (15%) and thrombocytopenia (2%). Five percent of patients experienced grade 4 neutropenia, with one febrile neutropenic episode requiring hospitalisation. Other grade 3 toxicities included neurotoxicity (5%), PPE (7%), stomatitis (5%) and allergic reaction (2%). There was one episode of exfoliative dermatitis (grade 4, 2%) and one episode of severe mucositis (grade 4, 2%). Total alopecia was noted in 72% of patients. Other nonhaematological toxicities were mild. There were no treatment-related deaths. Detailed toxicity data according to the NCI CTC criteria are presented in Table 3

**Table 3:** Treatment-related toxicity

	***N* (%)**			
**Toxicity**	**Grade 1**	**Grade 2**	**Grade 3**	**Grade 4**
Anaemia	9 (22)	6 (15)	6 (15)	—
Leukopenia	5 (12)	6 (15)	7 (17)	2 (5)
Neutropenia	2 (5)	4 (10)	5 (12)	2 (5)
Thrombocytopenia	—	—	1 (2)	—
Infection	2 (5)	1 (2)	—	1 (2)
Gastrointestinal (N/V)	10 (24)	1 (2)	—	—
Alopecia	2 (5)	30 (72)		
Neurotoxicity	5 (12)	3 (7)	2 (5)	—
Allergic reaction	1 (2)	1 (2)	1 (2)	—
Mucositis	4 (10)	3 (7)	1 (2)	1 (2)
Stomatitis	—	1 (2)	2 (5)	—
PPE	1 (2)	1 (2)	3 (7)	1 (2)

Febrile neutropenia occurred in one patient (2%). There were no treatment-related toxic deaths.

.

## DISCUSSION

The present study is the first to report on the efficacy and toxicity of a PLD-based combination in advanced STS, incorporating another new agent, paclitaxel. The response rates seen in our study are comparable to those of single-agent doxorubicin (Dirix and van Oosterom, 1999) and are definitively improved from those reported in PLD studies until now (Chidiac *et al*, 2000). Seven patients achieved objective responses, including one complete response, and a median TTP for responders of almost 6 months (TTP, 5.7 months; range, 1–52 months). In all, 15 patients progressed during treatment, while the remaining 14 patients achieved meaningful disease stabilisation. The median survival time was 13.2 months (range, 0.6–51.6 months), which compares to the highest survival times reported with standard regimens in advanced STS.

The doses and schedule of administration of the two drugs in our study were identified from previous phase I/II experience of our group and others (Briasoulis *et al*, 1999, Gogas *et al*, 2002). Although there is considerable experience with the combination of doxorubicin and paclitaxel, data regarding the use of PLD and taxanes are limited. Most studies in other tumour types where this combination has been evaluated, utilise paclitaxel at doses of 175 mg m^−2^ combined with 30–35 mg m^−2^ PLD, administered every 3–4 weeks (Briasoulis *et al*, 1999; Dirix and van Oosterom, 1999; Gogas *et al*, 2002). We opted for a higher dose of PLD (45 mg m^−2^) combined with paclitaxel at 150 mg m^−2^, and administration every 4 weeks, which has been shown to be the optimal time schedule for such doses with regard to skin toxicity. The above schedule exhibited a similar toxicity profile to reported combinations of paclitaxel–PLD in other tumour types (Briasoulis *et al*, 1999; Dirix and van Oosterom, 1999; Gogas *et al*, 2002). There was no grade 3 or 4 gastrointestinal toxicity, while myelosuppression was acceptable, with only one case of febrile neutropenia requiring hospitalisation. Skin and mucosal toxicities are the most frequent and prevalent toxicities of PLD administration. Similarly, these were the most common nonhaematological toxicities of the combination of PLD with paclitaxel in this group of patients, with 7% of patients experiencing grade 3 PPE and one patient discontinuing treatment due to exfoliative dermatitis. However, the incidence of marked skin toxicity observed in our study, is significantly lower than that usually reported when lower doses of PLD (35 mg m^−2^) are administered in shorter time intervals, every 3 weeks (Gogas *et al*, 2002). The addition of paclitaxel to the higher dose of PLD used in our study did not seem to affect the incidence of skin toxicity, as this is possibly related to the dose scheduling intervals and exposure to persistent drug levels (Lyass *et al*, 2000). There was no apparent cardiac toxicity observed in our cohort of patients. A 5% occurrence of grade 3 neurotoxicity, seen here, is consistent with other studies using similar doses of paclitaxel (Mavroudis *et al*, 2002). The administration of this regimen at the selected doses allowed for a successful delivery of the scheduled dose intensity.

The favourable prognostic factors for response to chemotherapy, as studied by the EORTC Soft Tissue and Bone Sarcoma Group, include young age, high grade, absence of liver metastases and liposarcoma histology (van Glabbeke *et al*, 1999). Our study included all STS histologies, no GIST, and a large majority of leiomyosarcomas (43%). The high proportion of leiomyosarcomas could account for the relatively low response rates obtained in our study, as some series suggest that this histological type is more chemoresistant than other STSs. The median age was that reported in most studies with STS (54 years), however, patients as old as 78 years of age were treated. The majority of patients (43%) had a visceral site of primary disease, with only 19% of primary extremity sites. A total of nine patients had received adjuvant chemotherapy after resection of primary disease, which was mainly anthracycline based. Although in all cases there was more than a 12-month interval between adjuvant chemotherapy and study treatment, previous exposure to anthracycline could lead to resistance and account as well for the relatively low response rates seen. Furthermore, at study entry, a high proportion of patients had distant metastatic disease (69%) with the commonest site being lungs (43%), however, 14% of the patients had liver involvement, which is considered an unfavourable prognostic factor for response, while more than one-third of the patients had extensive disease with multiple site involvement. This could possibly explain the lower than anticipated response rate seen in this study. As discussed by several investigators, the only way to avoid the large differences in response seen among studies in STS, is to focus on better stratification of patients with advanced STS according to prognostic factors.

In conclusion, the results of this study indicate that the novel combination of PLD with paclitaxel is a safe and well-tolerated regimen, demonstrating modest efficacy as first-line therapy in patients with advanced STS. Although the role of chemotherapy dose intensity in advanced STS is not fully established, it seems critical to combine the few active drugs at adequate doses and optimal schedules. Efforts to refine the currently available therapeutic options for patients with advanced STS in order to maximise the therapeutic index should continue together with basic research identifying critical targets and pathways for therapeutic intervention.
